# Spatial Organization of Chromatin: Transcriptional Control of Adaptive Immune Cell Development

**DOI:** 10.3389/fimmu.2021.633825

**Published:** 2021-03-29

**Authors:** Jagan M. R. Pongubala, Cornelis Murre

**Affiliations:** ^1^Department of Animal Biology, University of Hyderabad, Hyderabad, India; ^2^Division of Biological Sciences, University of California, San Diego, La Jolla, CA, United States

**Keywords:** chromatin organization, cis-regulatory interactions, gene regulatory networks, phase-separation, B and T cell development

## Abstract

Higher-order spatial organization of the genome into chromatin compartments (permissive and repressive), self-associating domains (TADs), and regulatory loops provides structural integrity and offers diverse gene regulatory controls. In particular, chromatin regulatory loops, which bring enhancer and associated transcription factors in close spatial proximity to target gene promoters, play essential roles in regulating gene expression. The establishment and maintenance of such chromatin loops are predominantly mediated involving CTCF and the cohesin machinery. In recent years, significant progress has been made in revealing how loops are assembled and how they modulate patterns of gene expression. Here we will discuss the mechanistic principles that underpin the establishment of three-dimensional (3D) chromatin structure and how changes in chromatin structure relate to alterations in gene programs that establish immune cell fate.

## Introduction

During the past two decades considerable progress has been made in the analysis of genes that code for transcription factors and signaling molecules that control the development of various hematopoietic cell lineages. Differentiation of a multipotent progenitor into committed adaptive immune cells involves the activation of cell type-specific genes and silencing of the expression of genes associated with alternative cell lineages (1, 2). There is a growing evidence that the 3D organization of the genome and chromatin folding is intimately associated cell fate decisions and function. Here, we review multiple levels of higher-order genome organization that orchestrate B and T cell development.

## Chromosome Territories – Heterochromatin and Euchromatin

**Figure 1:**
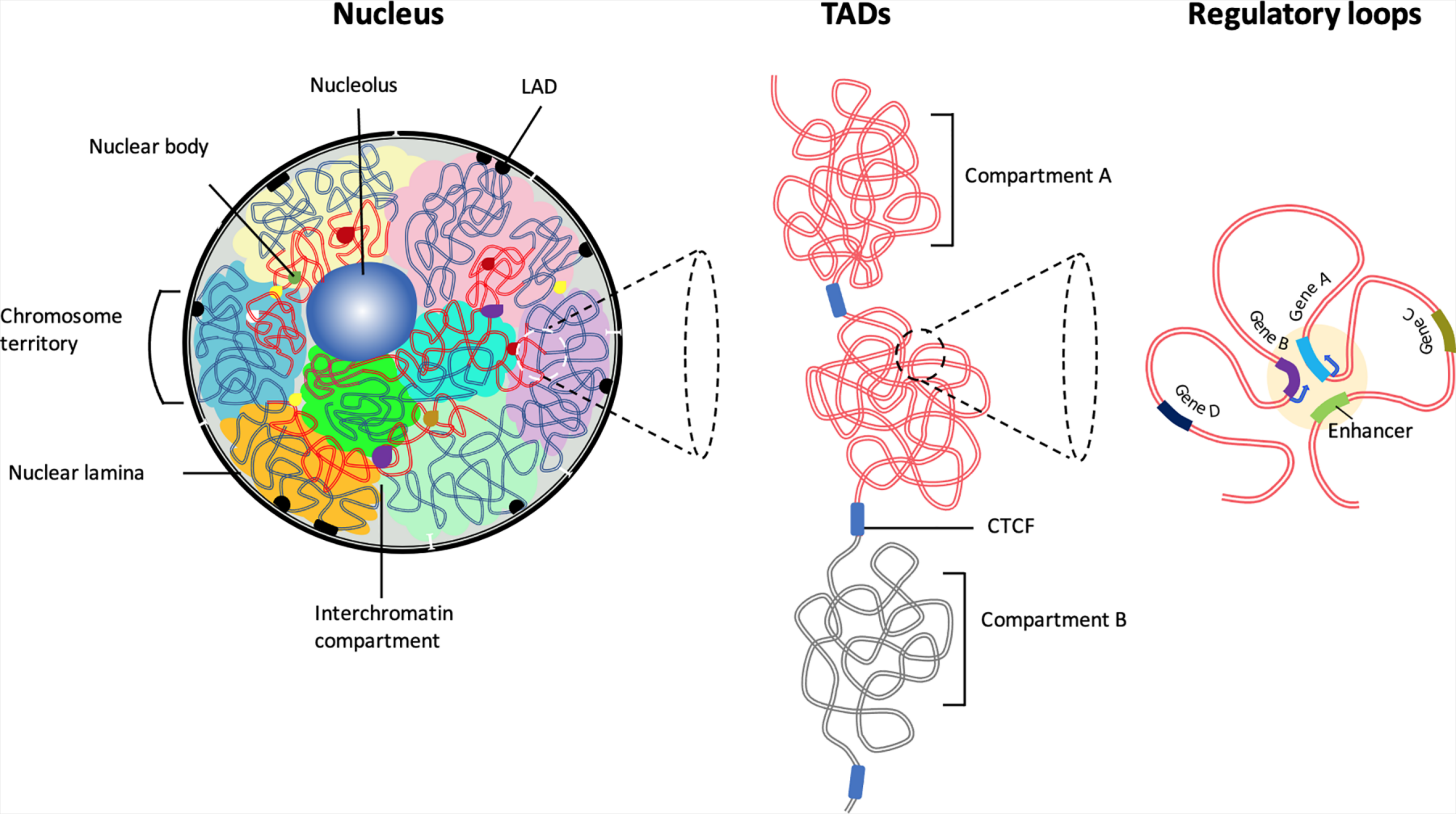
The hierarchical organization of the 3D chromatin. Inside the interphase nucleus, chromosomes occupy distinct territories (highlighted by different colors). Within each chromosome territory the chromatin is folded into multiple loops and segregated into two distinct compartments: compartment A clustered around nucleolus and nuclear bodies (permissive region, red lines), and compartment B (repressive region, blue lines) associated with LADs at the nuclear periphery. Chromatin interactions occur predominantly between the compartments with similar biochemical or functional properties. Majority of the chromatin interactions are intra-chromosomal. The permissive chromatin regions tend to position away from the nuclear lamina or from pericentromeric heterochromatin. Preferential self-interactions within the heterochromatin and euchromatin (A and B compartments) regions result in the formation of topologically associating domains (TADs), demarcated by boundary elements enriched with CTCF/Cohesin. Within TADs chromatin undergo multiple folds to form ‘regulatory loops’ that facilitate close proximity interactions between gene promoters and their cognate enhancers.

Although the nucleotide sequence of genomes of various mammalian cells have been revealed, the spatial arrangement of coding genes and associated regulatory elements within the confined three-dimensional (3D) space of the nucleus and its relation to the cell development and function remains unclear. It has long been assumed that chromosomes are sequestered into subnuclear structures such that complex biochemical reactions occur without crosstalk. Early microscopy studies revealed that in the interphase nucleus, each chromosome’s genome exists as a condensed unit in a distinct physical nuclear space termed as ‘chromosome territory’ (CT) (3–6). The position of chromosomes in 3D-nuclear space is nonrandom but is based on multiple features such as genomic length, gene-density, and transcriptional activity (**Figure 1**) (7, 8). Smaller chromosomes tend to be located toward the nuclear interior, whereas larger chromosomes are positioned near the nuclear periphery (9). Complementary biochemical approaches have demonstrated, wthin the CTs, chromatin is non-randomly folded as loops of varying genomic lengths and functionally segregated into euchromatin, consisting of open chromatin comprised of transcriptionally active regions, and heterochromatin containing highly condensed and transcriptionally repressed regions (8, 10–12) (**Figure 1**). While most of the gene activity of a specific chromosome is limited to its subnuclear space, some chromatin loops extend beyond the territory and engage in inter-chromosome interactions, creating an even distribution of the chromatin throughout the nuclear space (3, 12–15). These inter-chromosomal interactions not only regulate coordinated activation but also facilitate repression of gene expression patterns. Thus, chromatin-interaction networks within and between the CTs are non-randomly clustered to generate coregulated transcription hubs. These hubs utilize overlapping transcription factors and coactivators and function to facilitate increased transcription and transcript processing by associating with nuclear bodies, such as the nucleolus, Cajal bodies, and promyelocytic nuclear bodies (PML-NBs) (16, 17). Thus, nonrandom positioning of CTs generate distinct active and repressive genome neighborhoods.

## Gene Switching Between the Heterochromatic and Euchromatic Compartments

Genomic regions at the nuclear periphery predominantly interact with the lamina leading to the formation of higher-order structures known as lamina-associated domains (LADs) (**Figure 1**). Based on the enrichment of epigenetic marks and gene activity, LADs are located in transcriptionally repressive neighborhoods (18–20). In a recent study, it has been shown that promoters become active when moved away from their native LAD location (21). This is consistent with the notion that the physical association of lamina proteins, (LADs) impose major constraints on the shape and peripheral positioning of the genome and attenuation of transcriptional activity (20, 22). Selected genes in a repressive state at the nuclear periphery or heterochromatin regions detach and reposition into the nuclear interior and become transcriptionally active in a cell and developmental stage-specific manner (20, 23). Several examples highlight the functional relationship between transcriptional activity and genome topology during B and T lymphocytes development. For instance, lineage-specific antigen receptor rearrangement is closely associated with the ordered repositioning of these loci to a localized recombination center (24, 25). *IgH* locus rearrangements occur at the pro-B cell stage, whereas *Igκ* locus rearrangements occur at the small pre-B cell stage (26, 27). 3D imaging studies and chromatin conformation capture analyses indicate that prior to rearrangement, the Ig alleles are associated with nuclear lamina, a transcriptionally repressive compartment that inhibits somatic recombination. Preceding the pro-B cell stage *Igh* alleles move away from the nuclear lamina to the more central part of the nucleus, where they undergo large-scale conformational changes (28–30). As a result, the *Igh* proximal and distal Vh gene segments organize as loop domains close to the Dh segment (31–33). Successful V-D-J recombination of one of the *Igh* alleles results in the expression of the Ig μ heavy chain, which associates with surrogate light chain genes *(λ5* and *VpreB*) and leads to the assembly of the pre-BCR at the cell surface (30). Signaling through the pre-BCR complex blocks rearrangement of the other allele and instructs repositioning to the pericentromeric heterochromatin (24, 34–36). Conversely, at the pre-B cell stage, one of the *Igκ* alleles repositions to the pericentromeric heterochromatin allowing the other allele to undergo spatial rearrangement that favors recombination (34, 37, 38). Thus, the early-B cell developmental program is accompanied by the repositioning of Ig loci between euchromatin and heterochromatin compartments.

Similarly, Tcrβ and Tcrα loci undergo recombination during T cell development following relocalization and contraction by looping in a developmental stage-specific manner. The Tcrβ locus undergoes rearrangement in cells located in the double-negative (DN) compartment, whereas Vα-Jα rearrangements of the Tcrα locus occur during the double-positive (DP) cell stage (25, 39). The alteration in the spatial positioning of antigen receptor loci (β chain and α chain) has been suggested to provide accessibility to the recombination machinery (40–42). Notably, upon successful rearrangement of TCR alleles (β chain at DN stage or α chain at DP stage) followed by signaling through antigen receptors leads to a rapid reversal of locus contraction of non-productive alleles and their association with pericentromeric heterochromatin regions (40, 43–46). Nuclear repositioning and contraction of Ig and TCR loci are closely associated with the rearrangement, but the precise regulatory mechanisms that control these chromatin dynamics are not yet fully understood. Similarly, significant genome reorganization at the nuclear periphery and associated gene activity changes were detected during T cell development and function (22, 47). Thus, genome-nuclear lamina interactions are not only important for structural maintenance, but they are also intrinsically associated with gene regulation.

## Co-Regulatory Hubs

In addition to the dynamic alterations associated with the repositioning of genomic regions within CTs, nonrandom chromatin interactions are crucial for the overall nuclear organization of gene expression, where transcribing genes are clustered together at subnuclear sites enriched in transcription activators or silencing factors. A prominent example involves the expression of cytokines in peripheral T cells. Specifically in naïve T cells, the regulatory region of Th2 cytokine (encode IL-4, IL-5 and IL13), locus control region (LCR) located on chromosome 11, interacts with elements located across the interferon γ (*IFNγ*) gene located on chromosome 10 (48). These inter-chromosomal interactions have been suggested to be important for rapid transcription activation following stimulation of naïve T cells (49, 50). Upon terminal differentiation of naïve T cells into either Th1 or Th2 cells such inter-chromosomal interactions are lost (48). During Th2 cell development the LCR undergo a series of rapid epigenetic alterations following TCR stimulation and interacts with nearby regulatory elements to induce high levels of Th2 cytokine expression (51, 52). In Th1 cells, T-bet facilitates the interaction of the INFγ promoter with its enhancer to activate gene expression (48, 53). Th1, and Th2 cells repress *IL-4* and *IFNγ* gene expression by repositioning into heterochromatin regions (50, 54). Hence, the reciprocal pattern of *IL-4* versus *IFNγ* gene expression appears to be under the control of the transcription factors, T-bet and GATA-3 (55, 56).

## Switching Nuclear Location During Developmental Progression

Comparative Hi-C analysis of B cells has revealed that while during developmental progression the majority of the genes remain in the same compartments a small but significant percentage of genes (~10%) switched from compartment A to B and vice versa and displayed corresponding changes in transcript levels (57, 58). Prominent examples include *Ebf1, Satb2, Tead1, Pou2af1*, and *Tlr4*, reposition from compartment B to A during the developmental transition from pre-pro-B to pro-B cell stage. Relocalization of genes from compartment B to A resulted in a significant increase in promoter-enhancer interactions, leading to higher gene expression (57, 58). Conversely, genes such as *Satb1, cKit, Cd34* as well as crucial alternate cell fate determinants, including *Gata3, Zbtb16*, *Klf4, Vav3*, and *Sox6* relocate to compartment B at the pro-B cell stage (57, 58). Many essential alternate lineage determining factors (*GATA1, Gfi1, TCF7, Cebpα, Cebpβ, Bcl11b*, and *Id2*) are sequestered in a transcriptionally repressive compartment at the pro-B cell stage to ensure B cell-fate specification (58). Recent studies have revealed that plasma cell fate is orchestrated by widespread changes in nuclear architecture. In developing plasma cells the *Ebf1* locus is silenced by repositioning from euchromatic to the peri-centromeric heterochromatin region. Concomitantly, a distinct set of factors, including *Prdm1, Atf4*, and *Ell2* acquires the euchromatin state (59). Thus, gene repositioning positively correlates with transcriptional activity crucial for terminal plasma differentiation. These findings support the concept that genomic regions and single genes are nonrandomly arranged within the nucleus. Further supporting evidence for lineage determinants in the establishment of chromatin reorganization involves Bcl11b, a key regulator of T cell commitment. Activation of *Bcl11b* is contingent upon its interactions with its enhancer that binds Notch, GATA3, TCF1, and RUNX1 (60). Recent studies have shown that a long non-coding RNA known as thymocyte differentiation factor, ThymoD, repositions *Bcl11b* enhancer from a repressive compartment to an active compartment and juxtaposes the *Bcl11b* enhancer and promoter regions by forming a single loop domain (61). As expected, the absence of ThymoD results in a loss of activation of *Bcl11b* due to impaired recruitment of looping factors such as CTCF and the cohesin complex (61). Thus, selective gene activation or silencing during the developmental transition from multipotent progenitors to differentiated B and T cells is a recurrent principle that instructs adaptive immune cell development.

## Topologically Associating Domains

**Figure 2:**
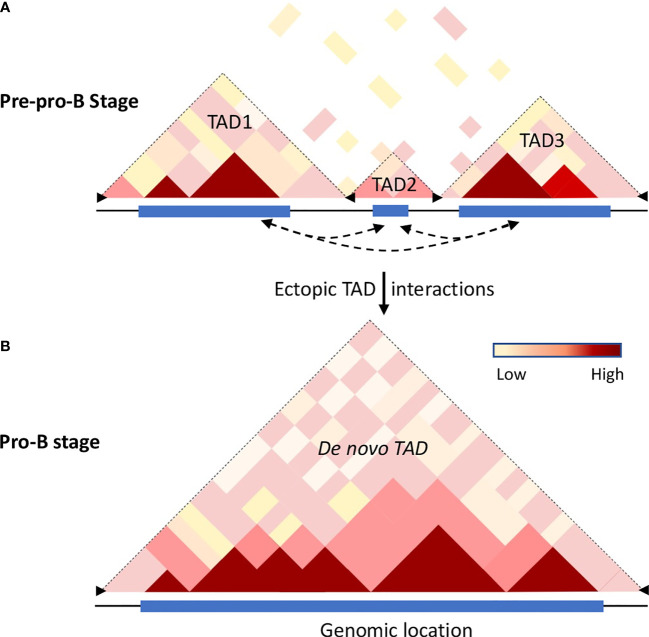
Chromatin dynamics in B cell progenitors. Hi-C analysis revealed that the mammalian genome is organized into topologically associated domains (TADs), generated by aggregation of self-interaction chromatin regions that feature similar properties, permissive or repressive, respectively. TADs are separated from each other by boundary elements that restrain interactions with adjacent TADs. The boundary elements are enriched with insulator proteins cohesin and CTCF. **(A)** The cartoon illustrates the Hi-C interactome of the mouse chromosome 12 for a genomic region that is partitioned into multiple TADs at the pre-pro-B cell stage. The frequency of intra-chromosomal interactions is indicated by the color gradient. **(B)** Schematic representation of the extended TAD of the same genomic region observed in committed B cells (57). During differentiation transcribing regions establish strong ectopic-TAD chromatin interactions leading to the merging of multiple adjacent TADs into a single *de novo* TAD. The increased cis-regulatory interactions within and between the TADs may be accomplished by loss of insulator proteins at the boundary regions accompanied by binding of TFs to their cognate regulatory elements thereby promoting alterations in genome confirmations. Dotted arrow lines represent possible ectopic-TAD interactions, blue rectangles represent genome scale high-frequency Hi-C interactions, and black arrowheads represent boundary elements.

It is now evident that the chromatin folds into clusters of loops, also named topologically associating domains (TADs) (**Figure 2**). TADs are stable and conserved across cell types (62–64). TADs are arranged contiguously across the chromatin and interspaced with boundary regions that are enriched for CTCF binding sites (62, 63, 65). It has been suggested that TADs ensure cell-type-specific gene expression by insulating the promoters from the enhancers located in a neighboring TAD and enriching for interactions between promoters and enhancers within TADs (5, 6, 10, 66, 67). Deletion of boundary sequences (63) or disruption of CTCF binding sites (68) readily result in alterations in TAD structure. Thus, TADs facilitate regulatory interactions while restricting interactions with genomic elements outside the loop domains (69). Consistent with these studies are observations indicating that genes positioned in the same TAD display similar expression patterns (57, 70).

## Dynamic TADs

Initial studies have indicated TADs are invariant structures, but the chromatin interactions within and between the compartments vary significantly (62, 65, 71). As described above, B and T cell development is closely associated with a number of chromatin alterations such as chromatin switching between permissive and repressive compartments, chromatin remodeling, and developmental stage-specific chromatin interactions beyond their chromosome territories. These observations suggest that genome structure and organization are highly dynamic and such alterations of chromatin topology may have a crucial role in genome function and maintenance of cell commitment. Hi-C analysis in differentiating B cells revealed that, while a substantial number of TADs are constant between pre-pro-B and pro-B cells, a significant number of TADs are altered (57). Based on the structural variations of TADs between pre-pro-B and pro-B cell stages, two distinct sets of TADs – unique TADs and dynamic TADs were identified. The unique TADs are those that are present only either in pre-pro-B or pro-B cell types. It has been proposed that increased local genomic interactions necessary for activation of lineage-specific genes may result in the formation of unique TADs. On the other hand, the dynamic TADs are those that undergo division or fusion during developmental progression (57). Indeed, a significant number of TADs (110) present at the pre-pro-B cell stage partitioned into two or more TADs at the pro-B cell stage. Conversely, a large number of TADs in the pre-pro-B cell stage coalesced to form extended TADs (183) at the pro-B cell stage (**Figure 2**). The division or merging of TADs may be associated with the loss or gain of inter-TAD interactions, respectively (57). Consistently, increased ectopic TAD interactions resulted in an expansion of median TAD size and a concomitant increase in promoter-enhancer interactions and high levels of transcription (57, 72). Thus, TADs are dynamic and undergo reorganization during developmental progression and disruption of boundary elements results in a regulatory loss or gain of gene activity leading to developmental abnormalities (**Figure 2**).

Recent investigations of genome mapping using single-cell Hi-C analysis and imaging studies have revealed that TADs may not prevail as constant units and may represent as collective chromatin interactome of a given cell population and thus challenges the conventional static view of topological chromatin domains (73–76). Although the single-cell Hi-C analysis corroborated the existence of large-scale chromatin compartments – permissive and repressive compartments, the self-interacting domains (TADs) were found to vary from cell-to-cell (72, 77). Concurrent with these observations, super-resolution imaging and single-cell Hi-C analysis demonstrated that the organizational structure, configuration, and boundaries of TADs vary extensively between individual cells (72, 73, 75, 78, 79). In line with these observations, biophysical studies indicate that cis-regulatory interactome differ dramatically from cell-to-cell (80). Contrary to population-average analyses (81, 82), high-resolution sequential chromatin tracing studies revealed TAD-like structural units in individual cells, despite depletion of cohesin, except the position of boundaries were altered significantly (73). It has been suggested that in the absence of cohesin, chromatin folds into loops of various length scales, as predicted by the chromatin-polymer model, resulting in the generation of globular structures involving intra-polymer interactions (83). Despite the nonrandom organization of chromosomes and genes, their localization and chromatin interactions vary from cell-to-cell, indicating that gene structure and activity are dynamic and stochastic (8, 74, 75). The heterogeneity of genome organization and function has been attributed to multiple influences, including extrinsic, allele-specific, and intrinsic factors (84). Extrinsic variability occurs due to cell-to-cell differences in the rate of transcription and differential expression levels of key transcription factors (85). In comparison, the intrinsic variability arises from differences in the binding of transcription factors to their cognate sites and dynamic chromatin movements in the 3D nuclear space (75). Finally, allele variability arises from the independent functioning of the two alleles in the same cell in a mutually exclusive manner (30, 35).

## Nuclear Architecture and Insulators

Numerous studies had now documented that depletion of cohesin (81, 86) or disruption of CTCF binding sites (87) readily results in impaired formation of loops and a reduction in the number of TADs. However, cohesin and CTCF do not play a significant role in segregating euchromatin from heterochromatin (68, 81, 82). Accumulating evidence indicates that in addition to CTCF other factors may also contribute to insulator function. For instance, the mediator complex functions in concert with cohesin to establish higher-order chromatin domains (88). Another transcriptional regulator, YY1, functions as a structural regulator of 3D genome (89). YY1 binds both promoters and active enhancers akin to that of CTCF. Disruption of YY1-binding motifs or deletion of YY1 impair enhancer-promoter looping and gene expression, indicating that YY1 is important for enhancer-promoter interactions (89). Earlier studies revealed that YY1 is essential for *Igh* locus contraction (90). Interestingly, like Pax5 deficiency (28), conditional deletion of YY1 results in a block at the pro-B cell stage and YY1-deficient pro-B cells fail to undergo *Igh* locus contraction and distal Vh-DhJh rearrangement (91). These findings have raise the question as to how these ubiquitously expressed factors (92–95) control cell-type-specific loop formation. It has been suggested that lineage-defining transcription factors instruct chromatin modifications and influence the establishment and maintenance of chromatin networks that promote lineage-specific gene expression program. Consistent with this possibility, *in situ* Hi-C analysis revealed that in developing B cells the genome topology undergoes widespread alterations involving cis-regulatory interaction landscape and that a majority of cis-regulatory elements bind Ebf1 and Pax5 (57). In line with these studies, a recent report shows that Pax5 plays an essential role in reorganizing the *Igh* locus contraction (28, 96). How do transcription factors modulate genome topology? It is possible that during B cell development transcriptional regulators initiate the formation of nuclear condensates. Indeed recent observations indicate that the *Igh* locus is organized as a solid or weak gel (97).

## Chromatin Loops

**Figure 3:**
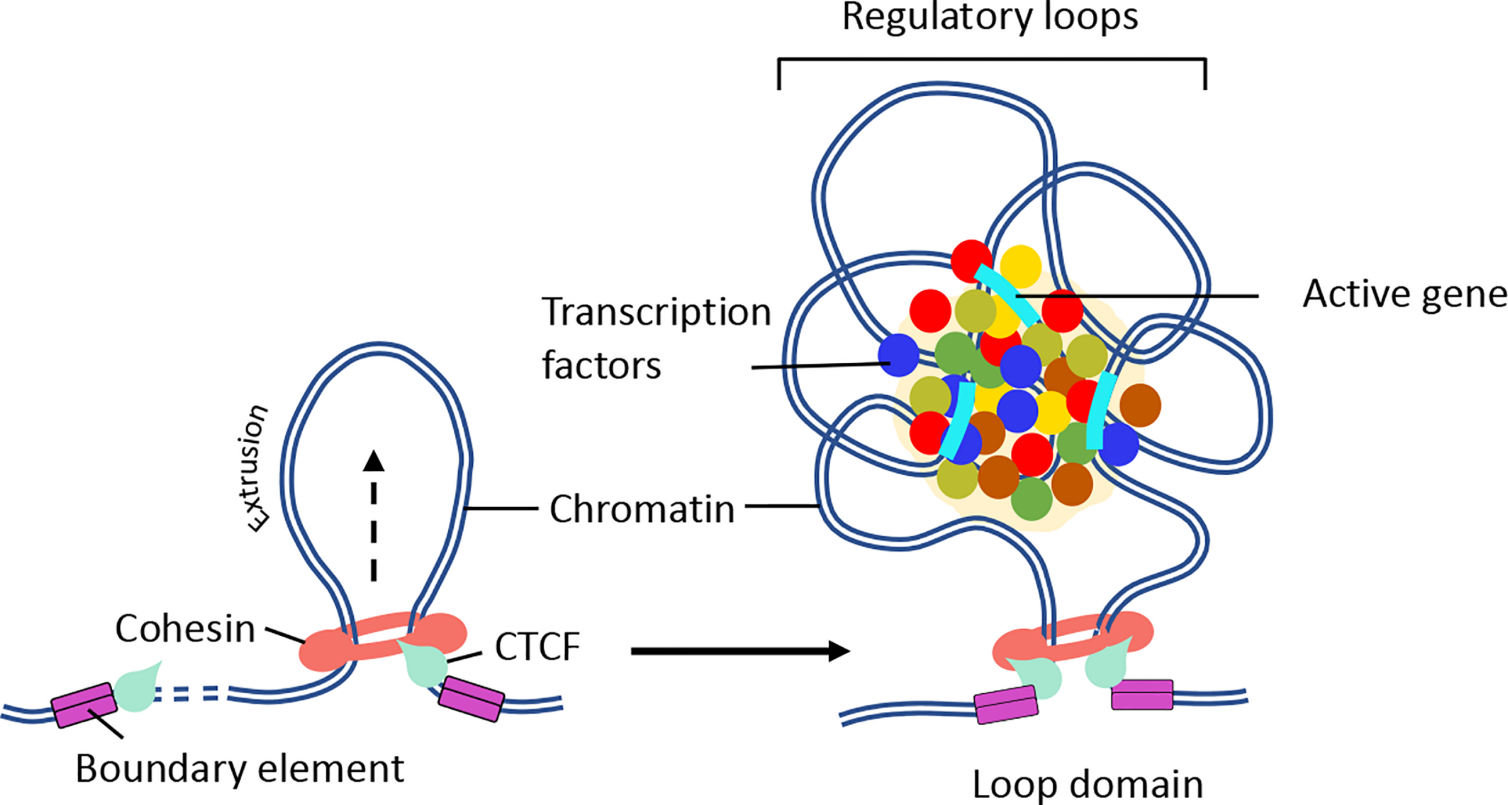
Mechanism of chromatin loop formation. TADs contain varying number of chromatin loops generated through loop extrusion by CTCF/cohesin complexes. Chromatin loop formation facilitates interactions between promoter and enhancer elements. (Right panel) In the presence of NIPBL and MAU2, the cohesin complex loaded on to the DNA. Then, cohesin extrudes chromatin until a pair of convergent CTCF binding sites is reached. (Right panel) The N-terminus of CTCF and convergent positioning of the CTCF-DNA complex stabilizes cohesin binding and stall chromatin extrusion leading to the establishment higher-order chromatin organization. The intervening DNA between two convergent CTCF sites leads to the formation of a loop domain, which adopts variety of complex shapes comprised of multiple regulatory loops. The internal structure of loop domain is likely determined by polymer chromatin-chromatin self-interactions, which may be further stabilized by phase separation. The contacts within the loop domains facilitate the targeting of enhancers to specific genes (104). The black arrow depicts the direction of loop extrusion.

It is now established that cohesin and CTCF function together to establish chromatin folding by loop extrusion (98–101). Briefly, cohesin is loaded onto chromatin by NIPBL and MAU2 heterodimer (102, 103). Once sequestered at chromatin, cohesin actively extrudes the chromatin fiber until it encounters two convergently bound CTCF sites (**Figure 3**) (87, 98, 101, 105, 106). The extruded loop then folds internally to form a large number regulatory loops, which promote interactions between regulatory elements and active genes within the same domain (**Figure 3**) (10). Cohesin is negatively regulated by WAPL, which dissociates cohesin rings from the chromatin (107, 108). Consistent with this function, deletion of cohesin releasing factor, WAPL, causes prolonged cohesin retention resulting in the enrichment of larger CTCF loops and decreased intra-TAD chromatin interactions (109, 110). Biophysical studies argue that the convergent CTCF barriers are important to ensure accurate genome folding (105, 111). Multiple regulatory loops often assemble into ‘transcription hubs’ where promoters and enhancers are spatially clustered (69). Contrary to the classical promoter-enhancer model (112), transcription hubs allow a single enhancer to co-activate multiple gene promoters. On the other hand, a single gene promoter can be co-activated by multiple enhancers (113–115). The transcription hubs recruit overlapping activating or repressing proteins through multiple mechanisms (**Figure 3**). Indeed synchronized transcription-bursting kinetics of the two spatially segregated genes was observed for a single enhancer (116). A recent study showed that recruitment of high concentration of activators may increase the distance between an enhancer and its target promoter upon transcriptional activation (117). The cis and trans physical contacts within these hubs may be established independently of CTCF/cohesin (68, 81, 118). In fact, disruption of the CTCF motif in the homeobox gene A (*HoxA)* locus leads to enhanced interactions between permissive and repressive regions and increased expression patterns (119). Another prominent example is where perturbation of CTCF-associated boundary elements results in increased activation of proto-oncogenes that are frequently associated with T-cell acute-lymphoblastic leukemia (T-ALL) (120). Similarly, inversion of CTCF motifs in the protocadherin alpha (Pcdha) locus led to expanded interactions between repressive and permissive hubs but led to decreased gene expression (121). These results indicate that CTCF boundary elements compartmentalize the genome into distinct domains and regulate gene expression by maintaining appropriate cis-regulatory interactions within the domain. Consistent with these possibilities, the majority of the TADs are composed of either permissive or repressive chromatin regions, as defined by histone modification patterns. However, only a small number of TADs are composed of both permissive and repressive regions (57).

## Nuclear Architecture and Antigen Receptor Locus Rearrangement

Antigen receptor locus rearrangement is regulated by enhancers as well as architectural proteins. The *Igh* locus contains multiple enhancers and particularly the intronic enhancer, Eμ, and the intergenic control region 1 (IGCR1) play crucial roles during locus contraction and rearrangement (122). Disruption of Eμ dramatically reduced Dh-Jh rearrangement in pro-B cells and thymocytes (123). Mutation of CTCF binding elements in IGCR1 disrupts the rearrangement order and displays severely reduced Vh utilization (124, 125). Similarly, the Igκ light chain contains two (iEκ and κE3’) enhancers. Deletion of either iEκ or κE3’ showed only a modest effect on Igk locus rearrangement. Conversely, deletion of both iEκ and κE3’ enhancers blocked the rearrangement of Igκ loci (126). In αβ T cells, successful rearrangement of TCRβ is under the control of Eβ enhancer (127). Similarly, the TCRα enhancer (Eα) is essential for double positive (DP) αβ T cell development (128). TCR locus rearrangement involving the TCR α, β, γ and δ loci is regulated by the helix-loop-helix proteins, E47 and HEB (129). In a recent study, it has been shown that E2A recruitment to distinct cis-regulatory elements regulates the expression of Rag-1 and Rag-2 genes, crucial for Ig and TCR gene rearrangements, in a lineage-specific manner (130). Thus various regulatory elements that bind distinct sets of transcription factors contribute to the spatial re-organization of the genome at a locus-specific level.

## The Role of Gelation or Phase Separation in Modulating Gene Expression

**Figure 4:**
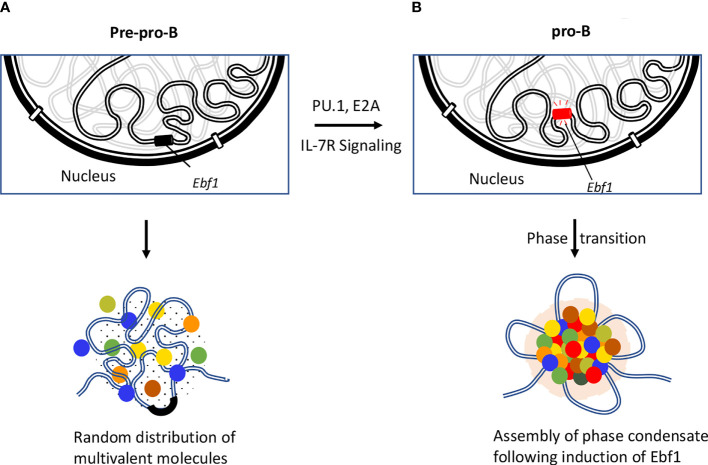
Role of EBF1 in orchestrating phase separated condensates. Repositioning of Ebf1 locus is illustrated during the developmental transition from pre-pro-B to pro-B stage. **(A)** Genomic locus of Ebf1 is localized in close spatial proximity to the repressive compartment of the nuclear periphery at the pre-pro-B cell stage. In the absence of Ebf1, B lineage genes are transcriptionally silent due to inaccessibility of the TF to their target sites or lack of TF that are necessary for their activation. **(B)** The Ebf1 locus repositions from the transcriptionally repressive compartment to the nuclear interior. The Ebf1 locus becomes transcriptionally active in response to PU.1 and E2A binding as well as IL-7R mediated signaling. It has recently been proposed that following activation of Ebf1 at pro-B cell stage and beyond, Ebf1 coordinates the establishment of multivalent interactions involving transcription factors, co-activators and transcription initiation proteins containing unstructured flexible regions to form a phase separation body (143).

While active genes move to euchromatin regions, the repressed genes are sequestered in highly condensed repressive regions, referred to as heterochromatin regions. Typically, heterochromatin regions comprised of repetitive sequences enriched for methylated histones (H3K9me3, H3K27me3), gene-poor regions and heterochromatin protein 1 (HP1) occupancy (131, 132). Recent studies revealed that assembly of heterochromatin domains is orchestrated by liquid-liquid phase separation (133, 134). Protein domains that are associated with modest structural complexity tend to assemble into high-density phase separated liquid condensates at clustered cis-regulatory elements (135–137). It has been proposed that transcription factors bind at enhancers and promoters to facilitate the formation of phase separation bodies by rapidly nucleating high concentration of activators, coactivators, and components of transcription initiation complex. Prominent among these factors is BRD4 a co-activator protein that is essential for the assembly of nuclear condensates involving super-enhancers (138). These phase separation bodies may play an important role in the assembly and function of eukaryotic genomes (136, 139, 140). Additionally, compartmentalization of the genome facilitates the assembly of enhancer clusters (super-enhancers) (141, 142). These findings led to the hypothesis that super-enhancers form phase separated biomolecular condensates akin to that described for the nucleolus and other membrane-less cellular bodies. The formation of phase separated bodies compartmentalizes and concentrates the transcription machinery to induce essential cell-identity genes (**Figure 4**) (137, 141, 142, 144). Biophysical studies suggest that transcription factors assemble into condensates (145). Most recent studies have indicated that the transcription factor EBF1 binds genomic regions prior to the detection of chromatin accessibility that requires the EBF1’s C-terminal domain (146). The Ebf1 C-terminal domains is interesting since it also harbors a prion-like domain with the ability to promote phase separation (143). Notably the ability to phase separate was significantly elevated by interaction of EBF1 with FUS, prion-like low-sequence complexity RNA-binding protein involved in transcription, DNA repair, and RNA biogenesis (147, 148). The chromatin remodeler Brg1, a key component of SWI/SNF chromatin-remodeling complex, also partitioned into phase separated FUS condensates and co-localized with EBF1 and FUS into distinct condensates (143). These elegant experiments revealed a pathway in which the phase separation ability of EBF1 facilitated Brg1-mediated chromatin opening. Finally, live cell imaging experiments recently revealed that remote genomic interactions are subject to severely sub-diffusive motion reflective of a solid or weak-gel configuration (97). Chromatin assembled into a solid or gel-like state facilitates remote genomic interactions within TADs assembly of droplets while preventing encounters with genomic regions located outside loop domains (97). It will be of significant interest to determine how transcription factors, histone acetyltransferases, and chromatin remodelers act in a solid or gel-like matter within the context of lymphoid specific patterns of gene expression and somatic recombination.

## Author Contributions

JP wrote the manuscript with inputs from CM. All authors contributed to the article and approved the submitted version.

## Conflict of Interest

The authors declare that the research was conducted in the absence of any commercial or financial relationships that could be construed as a potential conflict of interest.
